# Lymph Node Metastases of Medullary Thyroid Cancer: Role of Calcitonin in the Washout Fluid of Fine-Needle Aspiration

**DOI:** 10.1155/2020/9267972

**Published:** 2020-04-04

**Authors:** Bernardo Marques, Nuno Cunha, Raquel G. Martins, Ana Rita Elvas, Joana Couto, Jacinta Santos, Teresa Martins, Ana Paula Moniz, Olga Ilhéu, Frederico Valido, Fernando Rodrigues

**Affiliations:** ^1^Endocrinology Department, Instituto Português de Oncologia de Coimbra Francisco Gentil, EPE, Coimbra, Portugal; ^2^Laboratory Medicine Department, Instituto Português de Oncologia de Coimbra Francisco Gentil, EPE, Coimbra, Portugal; ^3^Pathology Department, Instituto Português de Oncologia de Coimbra Francisco Gentil, EPE, Coimbra, Portugal

## Abstract

**Introduction:**

The diagnostic value of calcitonin (CT) measurement in fine-needle aspirate washout (FNA-CT) for medullary thyroid cancer (MTC) lymph node (LN) metastases remains to be determined. It may increase the diagnostic sensitivity, but data on this subject is sparse.

**Objective:**

Our study aimed to evaluate the utility of FNA-CT in the diagnosis of LN metastases of MTC.

**Methods:**

We retrospectively investigated, in our institutional database, 69 consecutive FNA LN cytology from 42 patients who underwent FNA cytology and CT measurement in needle washout for suspicious LN between 2012 and 2017.

**Results:**

From the total of 69 FNA, 30 (43.4%) were performed in patients with personal history of MTC. MTC was detected in 19 FNA cytology (27.5%), and CT was detectable in needle washout in 23 cases (median = 2014 pg/mL; interquartile range = 490–15111 pg/mL). Based on the combined results of FNA-CT and FNA cytology, LN surgical resection was performed in 33 cases (47.8%). Histology reported MTC LN metastases in 21 lesions (63.6%). Regarding the diagnosis of MTC LN metastases, FNA cytology showed sensitivity of 81.8% and specificity of 97.9%, and FNA-CT demonstrated sensitivity of 100% and specificity of 97.9%. We determined through ROC analysis an optimal FNA-CT cut-off value of 23 pg/mL for the diagnosis of LN metastases (sensitivity 100%; specificity 100%).

**Conclusions:**

FNA-CT may be a valuable diagnostic tool for detection of MTC LN metastases, along with FNA cytology, and it should be included in the clinical workup of neck adenopathies in patients with MTC or with thyroid nodules.

## 1. Introduction

Medullary thyroid cancer (MTC) accounts for 1–3% of all cases of thyroid carcinoma and arises from parafollicular cells (C cells). About 20% of the cases are hereditary, as part of type 2 multiple endocrine neoplasia syndrome. At diagnosis, about 70–80% of the cases present with cervical lymph node (LN) metastases and 10% of cases also present with distant metastases [1–3].

All patients with a preoperative diagnosis of MTC should undergo neck ultrasound (US) and measurement of serum calcitonin (CT) [4]. Serum CT is a 32-amino acid linear polypeptide hormone, mainly produced by the C  cells, and it is regarded as a reliable MTC marker. When elevated in nonthyroidectomized patients, it should raise suspicion for its diagnosis and warrant complementary evaluation with fine-needle aspiration (FNA) cytology of suspicious thyroid nodules and cervical LN [5]. Thus, according to the American Thyroid Association (ATA) guidelines for the management of MTC, total thyroidectomy and dissection of cervical LN compartments, depending on serum calcitonin levels and neck US findings, is the standard treatment for patients with MTC [1].

There are a few other additional systemic and local therapies used to achieve tumor control, but their efficacy is limited. Therefore, complete surgical resection is of utmost importance for all MTCs and successful surgery with clear surgical margins, and removal of all LN metastases is a prerequisite for cure [1, 3].

During follow-up of MTC patients and in case of raising levels of serum CT, imaging exams should be performed in order to diagnose local recurrence, LN metastases, or distant metastases [1, 3]. LN metastases are usually diagnosed by neck ultrasound (US), as it is usually performed for the diagnosis of LN metastases of differentiated follicular thyroid carcinoma (DFTC). FNA cytology is an extremely useful method to confirm its diagnosis. However, the diagnostic accuracy of FNA cytology is lower in MTC than in DFTC (60–70% versus 90–95%) and heavily relies on the pathologists' experience [6, 7]. Typically, aspirates are hypercellular, composed of poorly cohesive cells, which are predominantly spindle shaped. Binucleation and eccentric nuclei as well as anisonucleosis and coarse chromatin are also present, and the cytological pattern may mimic papillary, anaplastic, or follicular carcinoma [6–8].

Patients with DFTC also have an additional tool for the diagnosis of LN metastases: measurement of thyroglobulin (TG) in FNA washout fluid. The ATA guidelines for the management of differentiated thyroid cancer recommend using both FNA cytology and measurement of TG in the washout fluid for confirmation of malignancy in suspicious LN, with a cut-off of <1  ng/mL for the exclusion of LN metastases [9]. Unlike DFTC, in which the measurement of TG is a well-established and reliable method for the diagnosis of LN metastases, in MTC, the data is sparse on the measurement of CT in FNA washout fluid (FNA-CT) of MTC LN metastases [9]. Few studies have been published on this subject, and they show that FNA-CT is a promising tool, as it has a higher accuracy for the diagnosis of metastatic LN than FNA cytology (60–70% versus 85–90%). However, most studies focus on this method for the diagnosis of MTC in both thyroid nodules and LN and the suggested cut-offs reflect this methodology. Besides, there are still many centers where this method is not applied and it is not recommended, yet, by the ATA guidelines for the management of MTC [1]. Our study aimed to further evaluate the utility of FNA-CT measurement exclusively in LN, in the diagnosis of MTC LN metastases and compare it with FNA cytology.

## 2. Materials and Methods

A retrospective analysis of our institutional database identified 69 consecutive FNA cytology and CT measurement in FNA washout of LN (from 42 patients) between 2012 and 2017. Measurement of FNA-CT in the washout of LN was performed in nonthyroidectomized patients evaluated in our department for suspicious neck LN and in patients with personal history of MTC, suspicious neck LN, and progressive increase in serum CT levels. In nonthyroidectomized patients, both TG and CT were measured in the LN washout fluid to exclude LN metastases from a thyroid carcinoma, and in patients with personal history of MTC, only CT was measured as an adjunct to cytologic examination. We did not measure FNA-CT in the washout fluid of thyroid nodules.

All patients underwent a thorough neck examination, neck US, and US-guided FNA of suspicious LN for conventional cytology and measurement of FNA-CT. Neck US was performed using a Canon Xario^TM^ 100 US machine, with a 4.2–14  mHz linear electronic transducer, by an endocrinologist with at least 5 years of experience. All LNs and cervical masses were identified and localized, and their diameters were measured. Suspicious LNs were identified according to standard criteria (loss of echogenic hilum, hyperechogenicity, cystic changes, calcifications, round shape, and abnormal vascularity) and submitted to one FNA cytology under US visual guidance with a 22 or 25-gauge needle attached to a 10  mL syringe [10]. The endocrinologist who performed the procedure excluded specimens that might have blood in the needle. A second FNA cytology was performed in case there was a doubt if there was no LN fluid in the needle.

Cytological examination was performed by two thyroid pathologists with at least 10  years of experience, and the diagnosis of MTC was made according to standard criteria [11].

After smear preparation, the syringe and needle were sent to the laboratory department to be flushed with 1  mL of normal saline solution (NaCl 0.9% m/V). The needle washout was centrifuged (1500  g, 2  min), and the supernatant, which is not the cellular material, was used for analysis. The solution was processed for FNA-CT measurement with a solid-phase, enzyme-labeled, two-site chemiluminescent immunometric assay (Siemens Healthcare Diagnostics, Llanberis, Gwynedd LL55 4EL, United Kingdom, Immulite 2000XPI Calcitonin), calibrated to the World Health Organization 2nd International Reference Preparation 89/620 standard. The limits of detection and quantification were 2.0 and 5.0 pg/mL, respectively, whereas the upper reference limits were 11.5 pg/mL for females and 18.2 pg/mL for males [12]. Serum CT was measured using the same assay.

Histological analysis of surgical specimens was made using standard pathological techniques, including detection of CT expression by immunohistochemistry.

In this study, we included demographic (age and gender), laboratory (serum CT and FNA-CT), clinical (age and gender), and pathologic data (results from FNA cytology and histological analysis of LN) extracted from patients' charts.

All statistical analysis was performed using SPSS Statistics 23.0 software (IBM Corp. Released 2015. IBM SPSS Statistics for Windows, Version 23.0. Armonk, NY) Parametric statistical tests (*t*-tests) were used for variables following a normal distribution. The Mann–Whitney test was used when these premises were not met. For the categorical variables, we used the chi-square test and Fisher's exact test. To determine the best cut-off value for washout fluid FNA-CT, we used the receiver operating characteristic (ROC) analysis.

## 3. Results

This study's population had a mean age of 60.7 ± 15.9 years and was mainly comprised of male patients (*n* = 24 (57.1%)). Out of a total of 69 LN FNA cases, 39 (56.5%) were performed in nonthyroidectomized patients evaluated in our department for suspicious neck LN. The remaining 30 cases were performed in patients with personal history of MTC, suspicious neck LN, and progressive increase in serum CT levels. Cytologic examination detected MTC in 19 FNA (27.5%) and DFTC in 11 FNA (15.9%). Thirty-five (50.7%) FNA were diagnosed as benign and 4 (5.8%) were nondiagnostic.

**Figure 1:**
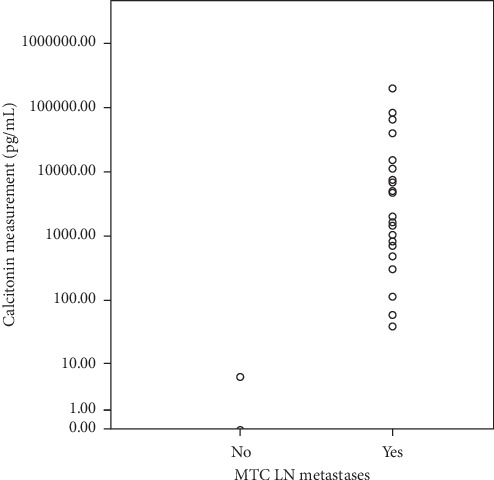
Distribution of MTC LN metastases according to FNA-CT measurements.

Calcitonin was detectable in needle washout in 23 (33.3%) FNA. In patients with detectable levels of FNA-CT, median was 635 pg/mL (interquartile range 277–2524). These patients had a median serum CT level of 2014 (490–15111) pg/mL and a mean LN diameter of 19.7 ± 10.7 mm. These results are shown in Tables 1 and 2 and Figure 1.

Very high levels of FNA-CT (>200 pg/mL) were seen in most cases (*n* = 19) and only 4 cases had needle washout CT levels below 100 pg/mL. Ten patients had two measurements of FNA-CT in the same lymph node, and 4 patients had FNA-CT measurements on different LN. Seven of the 23 detectable measurements of FNA-CT in LN washout fluid had higher levels of serum CT, and all of these cases had histologically proven MTC LN metastases (Table 2).

Lymph node resection was performed in 17 patients (36 lesions, 52.1%): 9 patients (24 lesions, 34.7%) with detectable levels of FNA-CT and 8 patients (12 lesions, 17.3%) with undetectable FNA-CT. Patients with undetectable FNA-CT underwent surgery for Bethesda V on thyroid nodule cytology and LN cytology suspicious for DFTC LN metastasis. Lymph node excisional biopsy was additionally performed in 7 patients with suspicious LN on neck US but undetectable levels of FNA-CT and benign cytology. Histological examination of these LN proved them to be granulomatous disease.

**Figure 2:**
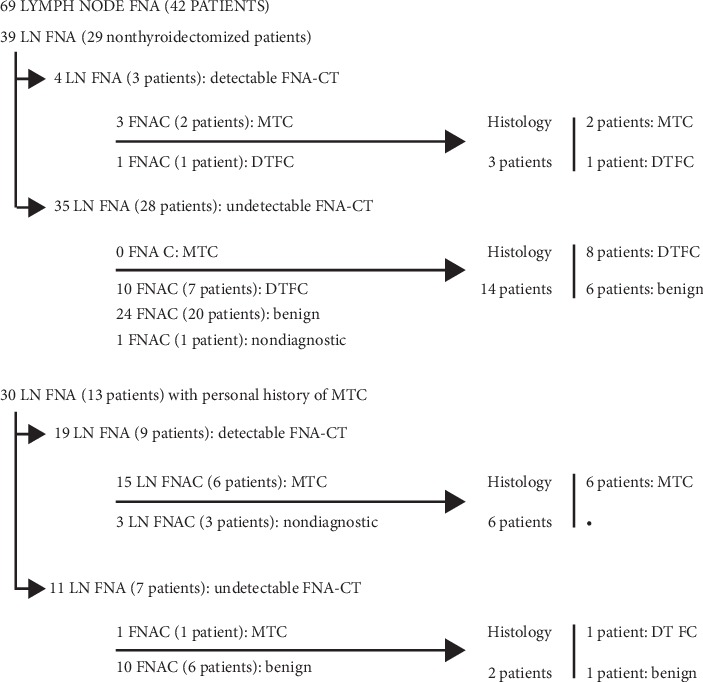
Histologic examination of LN, according to FNA-CT levels.

Histology reported LN metastases of MTC in 8 patients (19 lesions, 52.8%) and LN metastases of DFTC in 10 patients (12 lesions, 33.3%). All but one patient with DFTC had very low levels of FNA-CT, and it was the only false-positive (FP) in our study. He had a FNA-CT level of 5.6 pg/mL, a FNA cytology suspicious for papillary thyroid carcinoma, and a serum CT of 7 pg/mL. There was only one case of positive FNA-CT (1488 pg/mL) and MTC LN metastases on cytologic examination, without histological confirmation. It was a patient with known progressive MTC with two previous lateral-cervical dissections. He was diagnosed with LN recurrence and pulmonary metastases, and it was decided not to perform an additional LN dissection, but to start systemic therapy instead. These results are shown in Figure 2.

Regarding the diagnosis of MTC LN metastases, FNA-CT showed sensitivity of 100%, specificity of 97.9%, positive predictive value (PPV) of 95.6%, and negative predictive value (NPV) of 100%. There was only one FP, as was previously mentioned. On the other hand, FNA cytology had a sensitivity of 81.8%, specificity of 97.9%, PPV of 94.7%, and NPV of 92%. This method had only one FP, which was a patient with personal history of MTC with FNA-CT of <2 pg/mL, in which the pathology report confirmed no signs of malignancy.

FNA cytology was considered nondiagnostic in four cases (5.8%). Three of them had personal history of MTC and positive FNA-CT levels (312 pg/mL, 704 pg/mL, and 1086 pg/mL, respectively). They also had rising levels of serum CT, which increased the suspicion of metastatic disease. In these cases, high levels of FNA-CT in the washout lead to the diagnosis of LN metastases. Surgery was later performed, which confirmed the diagnosis of MTC LN metastases by histological analysis.

Combining both diagnostic methods showed the highest diagnostic power (sensitivity of 100%, specificity of 97.9%, PPV of 95.6%, and NPV of 100%). The lowest value of FNA-CT with histologically proven MTC LN metastases was 40.4 pg/mL. The optimal cut-off value of FNA-CT for determination MTC LN metastases through ROC analysis was 23.0 pg/mL (area under the curve = 99%; *P* < 0.001), leading to a sensitivity of 100% and specificity of 100%. These results are show in Table 3.

## 4. Discussion

Cytologic evaluation of suspicious LN is the most used diagnostic tool for detection of MTC LN metastases [1, 8, 13–16]. However, its accuracy is not as high as in DFTC. In this study, we assessed the potential utility of FNA-CT alone or combined with cytology, in the diagnosis of MTC LN metastases, as it is usually performed for DFTC. Studies regarding this subject suggest that this method might be an effective auxiliary method to FNA cytology, with sensitivity ranging from 89–100%. However, in most of these studies, FNA-CT is measured in either thyroid nodules or in both thyroid nodules and suspicious neck LN. Hence, suggested cut-offs for the diagnosis of MTC are usually the same in both situations. The ideal cut-off for the diagnosis of MTC in thyroid nodules or neck LN is estimated to be 10–30 pg/mL. This is the first study that analyzed the utility of FA-CT measurement exclusively in MTC LN metastases and has the largest cohort of LN.

Our results showed that high levels of FNA-CT were present in all patients with histologically proven MTC with a sensitivity of 100% and specificity of 97.9%. The lowest value of FNA-CT in a histologically proven metastatic LN was 40.4 pg/mL, and the optimal cut-off, according to ROC analysis is 23.0 pg/mL, which is similar to other cut-offs proposed by other studies [8, 12, 13, 15, 16]. The only FP was a patient with a low value of FNA-CT and serum CT. This patient was submitted to surgical LN dissection and the pathology report revealed LN metastases of DFTC. In this case, it might have been a laboratory error, as the FNA-CT level fell near the functional sensitivity range.

There was a wide range of FNA-CT measurements between patients, as well as high variability between measurements in the same LN. This can be explained by the fact that each fine-needle aspiration is a different procedure in and of itself and bound to get a different amount of LN fluid, each time it is performed. The wide range of FNA-CT values in different LN might be due to the inherent differences in calcitonin secretion by different tumors. We would like to point out that, despite of the broad range in FNA-CT measurements, the diagnostic accuracy is very high and we had no false-negatives and only one false-positive.

Fine-needle aspiration cytology also showed high accuracy for the diagnosis of LN metastases and higher than previous studies (sensitivity of 81.8% and specificity of 97.9%) [12, 13, 16]. This can be attributed to the significant experience our pathologists have earned through the years, working in an oncology center with a large number of cases of both DFTC and MTC and to the fact that they were not blinded to the results of FNA-CT measurements.

Some studies also evaluated the potential utility of the FNA-CT/serum CT ratio for the diagnosis of MTC in thyroid nodules and LN, which could be useful in order to avoid false elevations of CT in the washout fluid if serum CT is significantly elevated. In order to avoid this potential bias, we excluded cases that might have had blood in the needle and all of them were centrifuged to ensure that only the supernatant was analyzed. We also need to keep in mind that the aspirate leftover volume is usually very small and subsequently rinsed/diluted with 1  mL of normal saline solution to overcome possible interferences of serum CT in FNA-CT measurement. Thus, we did not find the ratio FNA-CT/serum CT useful in this situation. In fact, 22 of the 23 detectable measurements were true-positives and only 7 of them had higher levels of serum CT than FNA-CT. All of these cases had histologically proven MTC LN metastases.

As with most clinical studies of thyroid cancer, this is a retrospective study and potential selection bias should be considered in the interpretation of the results. MTC is a rare entity and this study has a heterogeneous cohort. On the other hand, there is still limited evidence regarding CT measurement in FNA washout fluid. Therefore, in this context, we believe that it is important to evaluate and describe the results from clinical practice.

## 5. Conclusion

This is the largest study regarding the diagnostic utility of FNA-CT for detection of MTC exclusively in LN. It further supports the value of FNA-CT as a reliable and inexpensive diagnostic tool, along with FNA cytology, that should be included in the clinical workup of neck adenopathies in patients with thyroid nodules or medical history of MTC. However, there is still need for further studies in order to assess the actual relevance of this technique in the management of MTC and validate the optimal cut-off value for the diagnosis of LN metastases.

## Figures and Tables

**Table 1:** Patients characteristics according to FNA-CT measurement.

		FNA-CT measurements *N* = 69 (42 patients)		*P*
		Detectable *N* = 23 (10 patients)	Undetectable *N* = 46 (32 patients)	
Gender	Female	4	15	NSS
	Male	6	17	

Age (years)		63.2 ± 16.7	57.9 ± 14.2	NSS
Personal history of MTC		7	6	NSS
FNA-CT levels (pg/mL)^*∗*^		2014 (490 – 15111)	<2 (0)	<0.001
Serum CT (pg/mL)^*∗*^		635 (277 – 2524)	1 (1 – 277)	0.016
LN diameter (mm)		19.7 ± 10.7	16.2 ± 6.1	0.014

FNA-CT levels and serum CT levels expressed as median (interquartile range); age and LN diameter expressed as mean ± standard deviation; NSS = no statistical significance.

**Table 2:** Patients with detectable levels of FNA-CT in LN washout fluid (available as a supplementary file).

	Personal history	LN (size, mm)	FNAC	FNA-CT (pg/mL)	Serum CT (pg/mL)	Histology
Patient 1	MTC	LN1 (18)	MTC LN metastasis	200000	86.9	MTC LN metastasis
Patient 2	MTC	LN 1 (17.4)	Nondiagnostic	704	932	MTC LN metastasis
Patient 3	MTC	LN 1 (15.5)	Nondiagnostic	1086	533	MTC LN metastasis
Patient 4	MTC	LN1 (15)	Nondiagnostic	312	9704	MTC LN metastasis
Patient 5	MTC	LN1 (16)	MTC LN metastasis	85621	635	MTC LN metastasis
Patient 6	MTC	LN 1 (25)	Benign	<2	794	—
				<2		
		LN 2 (40)	MTC LN metastasis	11065	3796	MTC LN metastasis
				40226		
		LN3 (15)	MTC LN metastasis	4913	121	MTC LN metastasis
		LN 4 (12)	Benign	<2	340	—
				<2		
Patient 7	Nonthyroidectomized	LN 1 (45)	MTC LN metastasis	15111	1871	MTC LN metastasis
	MTC	LN 2 (14)	MTC LN metastasis	6779	1224	MTC LN metastasis
				60.2	1224	
Patient 8	MTC	LN 1 (13.2)	MTC LN metastasis	117	277	MTC LN metastasis
				2014		
		LN 2 (18.8)	MTC LN metastasis	4653		MTC LN metastasis
		LN 3 (12)	Benign	<2	545	—
				<2		
		LN 4 (14)	Benign	<2		—
		LN 5 (18)	MTC LN metastasis	40.4	598	MTC LN metastasis
				490		
		LN 6 (13)	MTC LN metastasis	838	515	MTC LN metastasis
				1686		
Patient 9	Nonthyroidectomized	LN 1 (25.5)	DTFC	5.6	7	DFTC LN metastasis
Patient 10	Nonthyroidectomized	LN 1 (25)	MTC LN metastasis	7364	5007	MTC LN metastasis
				66104		
	MTC	LN 2 (28.4)	MTC LN metastasis	200000	1355	MTC LN metastasis
		LN 3 (27)	MTC LN metastasis	1488	2524	^*∗*^

^*∗*^One patient with a detectable FNA-CT (1488 pg/mL) and MTC LN metastases on cytologic examination did not undergo surgery and started systemic therapy.

**Table 3:** Diagnostic accuracy of FNA cytology and FNA-CT.

	MTC	Non-MTC	Nondiagnostic	Sensitivity (%)	Specificity (%)	PPV (%)	NPV (%)
FNA cytology	19 (27.5%)	46 (66.6%)	4 (5.8%)	81.8	97.9	94.7	92.0
FNA-CT	23 (33.3%)	46 (66.6%)	—	100	97.9	95.6	100
FNA cytology + FNA-CT	23 (33.3%)	46 (66.6%)	—	100	97.9	95.6	100

## Data Availability

The clinical data used to support the findings of this study are included within the article.

## References

[1] Wells S. A., Asa S. L., Dralle H. (2015). Revised American Thyroid Association guidelines for the management of medullary thyroid carcinoma. *Thyroid*.

[2] Kim B. H., Kim I. J. (2016). Recent updates on the management of medullary thyroid carcinoma. *Endocrinology and Metabolism*.

[3] Moley J. F., DeBenedetti M. K. (1999). Patterns of nodal metastases in palpable medullary thyroid carcinoma. *Annals of Surgery*.

[4] Kouvaraki M. A., Shapiro S. E., Fornage B. D. (2003). Role of preoperative ultrasonography in the surgical management of patients with thyroid cancer. *Surgery*.

[5] Yip D. T., Hassan M., Pazaitou-Panayiotou K. (2011). Preoperative basal calcitonin and tumor stage correlate with postoperative calcitonin normalization in patients undergoing initial surgical management of medullary thyroid carcinoma. *Surgery*.

[6] Suzuki A., Hirokawa M., Takada N. (2017). Fine-needle aspiration cytology for medullary thyroid carcinoma: a single institutional experience in Japan. *Endocrine Journal*.

[7] Canberk S., Firat P., Schmitt F. (2015). Pitfalls in the cytological assessment of thyroid nodules. *Turkish Journal of Pathology*.

[8] Siqueira D., Rocha A. P., Puñales M. K., Maia A. L. (2009). Identification of occult metastases of medullary thyroid carcinoma by calcitonin measurement in washout fluid from fine needle aspiration of cervical lymph node. *Arquivos Brasileiros de Endocrinologia & Metabologia*.

[9] Haugen B. R., Alexander E. K., Bible K. C. (2016). 2015 American Thyroid Association management guidelines for adult patients with thyroid nodules and differentiated thyroid cancer: The American Thyroid Association Guidelines Task Force on Thyroid Nodules and Differentiated Thyroid Cancer. *Thyroid*.

[10] Leenhardt L., Erdogan M. F., Hegedus L. (2013). 2013 European Thyroid Association guidelines for cervical ultrasound scan and ultrasound-guided techniques in the postoperative management of patients with thyroid cancer. *European Thyroid Journal*.

[11] Cibas E. S., Ali S. Z. (2009). The Bethesda system for reporting thyroid cytopathology. *American Journal of Clinical Pathology*.

[12] Trimboli P., Cremonini N., Ceriani L. (2014). Calcitonin measurement in aspiration needle washout fluids has higher sensitivity than cytology in detecting medullary thyroid cancer: a retrospective multicentre study. *Clinical Endocrinology*.

[13] Boi F., Maurelli I., Pinna G. (2007). Calcitonin measurement in wash-out fluid from fine needle aspiration of neck masses in patients with primary and metastatic medullary thyroid carcinoma. *The Journal of Clinical Endocrinology & Metabolism*.

[14] Kihara M., Hirokawa M., Kudo T. (2018). Calcitonin measurement in fine-needle aspirate washout fluid by electrochemiluminescence immunoassay for thyroid tumors. *Thyroid Research*.

[15] Baldini E., Sorrenti S., Di Gioia C. (2013). Cervical lymph node metastases from thyroid cancer: does thyroglobulin and calcitonin measurement in fine needle aspirates improve the diagnostic value of cytology?. *BMC Clinical Pathology*.

[16] Bugalho M. J. M., Santos J. R., Sobrinho L. (2005). Preoperative diagnosis of medullary thyroid carcinoma: fine needle aspiration cytology as compared with serum calcitonin measurement. *Journal of Surgical Oncology*.

